# What Makes Urban Communities More Resilient to COVID-19? A Systematic Review of Current Evidence

**DOI:** 10.3390/ijerph191710532

**Published:** 2022-08-24

**Authors:** Peng Cui, Zhiyu Dong, Xin Yao, Yifei Cao, Yifan Sun, Lan Feng

**Affiliations:** Department of Engineering Management, School of Civil Engineering, Nanjing Forestry University, Nanjing 210037, China

**Keywords:** urban communities, COVID-19, social resilience, systematic literature review

## Abstract

It has been more than two years since the outbreak of the COVID-19 epidemic at the end of 2019. Many scholars have introduced the “resilience” concept into COVID-19 prevention and control to make up for the deficiencies in traditional community governance. This study analyzed the progress in research on social resilience, which is an important component of community resilience, focusing on the current literature on the impact of social resilience on COVID-19, and proposed a generalized dimension to integrated previous relevant literature. Then, VOSviewer was used to visualize and analyze the current progress of research on social resilience. The PRISMA method was used to collate studies on social resilience to the pandemic. The result showed that many current policies are effective in controlling COVID-19, but some key factors, such as vulnerable groups, social assistance, and socioeconomics, affect proper social functioning. Some scholars have proposed effective solutions to improve social resilience, such as establishing an assessment framework, identifying priority inoculation groups, and improving access to technology and cultural communication. Social resilience to COVID-19 can be enhanced by both external interventions and internal regulation. Social resilience requires these two aspects to be coordinated to strengthen community and urban pandemic resilience.

## 1. Introduction

Since December 2019, the COVID-19 pandemic has persisted worldwide for more than two years. The virus not only has a simple transmission route, a long incubation period, and the ability to infect many close contacts, it is also highly capable of spreading and causing concentrated outbreaks. Infected people experience irreversible damage to the lungs and nerves, psychological and cognitive impairment, and even death. During this two-year period, COVID-19 continues to mutate while causing harm, resulting in an pandemic. As of 1 July 2022, a cumulative total of 544 million people had been diagnosed and 6.34 million people had died worldwide. The pandemic had affected more than 208 countries, causing far-reaching effects and enormous damage.

From a temporal perspective, after the first outbreak of COVID-19 in China, the Chinese government took emergency measures to control the spread of the epidemic and achieved a milestone victory. As the epidemic gradually expanded to Central and East Asia, then to Europe, South Asia, and the Americas, several countries began taking border control and blockade measures. The World Health Organization (WHO) defined COVID-19 as a pandemic on March 11. For example, Mongolia was the first to respond and enacted a lockdown policy [1]; Spain declared a state of emergency and adopted a mandatory confinement policy [2]; Italy issued an emergency control policy to break the cycle of infection and limit the spread of the outbreak [3]; Iran used continuous monitoring of information to seek emergency relief from the WHO to avoid further spread [4]; Canada closed borders to prevent offshore importation of the disease; and South Africa implemented health control measures to slow COVID-19 and co-infections of other infectious diseases [5]. Worldwide, the presence of COVID-19 seriously jeopardizes the work and life of social groups with a high probability of exposure to COVID-19, especially health care workers, while the economic, political and cultural development of countries is severely hampered [6,7,8,9,10]. COVID-19 often causes severe sequelae, psychological effects, and permanent damage in infected individuals, such as erectile dysfunction, orbital compartment syndrome, and focal status epilepticus [11,12,13]. It is a long-term disease like AIDS [14]. Therefore, determining an adaptive plan to prevent and control the epidemic in a timely, effective, and accurate manner is an important issue.

The related policies have been introduced at the national level and gradually advanced to the provinces. In the management of cities, control of COVID-19 has been achieved by dividing the area into communities to form smaller areas of activity.

The concept of “community” is recognized as having been first introduced by the German sociologist Tonnes in 1887. The concept first appeared in this work, which is an important marker for the inclusion of communities in sociological research [15]. A community is a group of people guided by shared emotions and values, developed through their own natural will and through their neighbors. In a community, people live in close but relatively narrow relationships with each other. The community is the smallest unit of the national governance system, and the community is not only the front line in fighting COVID-19, but also the link to coordinate between the government and the residents, i.e., from the country to the province/state, then to the city, and finally to the community. Taking the community as the unit, the government used a grid to divide its jurisdiction to form a smaller management scope, to achieve the prevention and control of COVID-19 from the “bottom up” [16,17]. Gu indicated that during the first outbreak of COVID-19 in China, the neglect of community governance led to the failure of early warnings [18]. Zaman revealed that, instead of top-down command and control, the Bangladeshi state relied on slum dwellers to respond spontaneously with strong informal interventions that worked well [19]. Wang emphasized the significant role played by community governance in improving community constructions in the face of large-scale public health emergencies [20]. Enhanced community governance and social support systems facilitate the resolution of intra-community conflicts and increase resistance to COVID-19 [21].

Resilience is defined as the distinctive elements of resilience, including the amount of disturbance a system can absorb while remaining within the same state [22], and the degree to which the system is capable of self-organization [23]. The resilience can be invoked in community governance as an important direction that takes into account both the context and the adaptive capacity of the system [24]. Social resilience focuses on community resilience in terms of overall social systems to prepare for disasters and reduce risk. The social resilience of groups and communities, as a component of community resilience to epidemics, has received attention from numerous researchers. Scholars have argued that the relationship between the impact of social resilience on COVID-19 is bidirectional, with social resilience providing value to emergency treatment, and epidemics such as COVID-19 promoting population safety and health programs that increase social resilience [25]. Leveraging social resilience can help accelerate the achievement of dynamic clearances and effective proactive prevention and control measures [26]. In the context of COVID-19, social resilience is increased through knowledge and social learning activities that provide protection to the population and potentially help community preparedness efforts [27]. Social resilience plays an important role in neighborhood coordination, and neighborhoods maintain a dynamic pattern of cooperation that accelerates control over COVID-19 [28]. COVID-19 control efficiency can be accelerated by rapidly establishing dedicated facilities to achieve increased levels of social resilience [29]. Thus, the development of resilience at the community level has very important implications for the public health variables and the health care services of individuals and societies [30,31].

In summary, analyzing epidemic prevention and control from the perspective of social resilience can not only accurately block the spread of the epidemic, but also quickly mitigate and minimize the negative impact of the epidemic on a larger regional scale. The related research on epidemic resilience involves dimensions, influencing factors, evaluation indicators, etc. However, previous studies have not included a review article in this field. In addition, the direction and dimensions of their analyses differ due to the different development and environmental issues, making research in this direction fragmented [32]. Therefore, there is a need to focus and analyze the development of social resilience, and the manner in which social resilience in the COVID-19 context helps communities to fight COVID-19, so that the relevant literature does not continue to be fragmented.

## 2. Materials and Methods

The Preferred Reporting Items for Systematic Reviews and Meta-Analyses (PRISMA) statement method was used to select and analyze studies of social resilience governance [33]. The specific steps were as follows.

(1) The articles in this study were collected from Web of Science, Scopus, and Springer Link, the main databases of global scientific articles. The keywords were set as “social resilience” AND “epidemic” (OR “pandemic”) (for the years 2012 to 2022) and set as “social resilience” (OR “social vulnerability”) AND “COVID-19*” (OR “coronavirus*” OR “SARS-CoV-2 virus”) (for the years 2019 to 2022). After removing duplicates, the search scope was limited to full-text published articles in English academic journals.
(2) VOSviewer software was used to visualize the articles’ “social resilience” AND “epidemic” (OR “pandemic”) (2012–2022). The articles were used as a data source for the knowledge mapping analysis after suggesting material, corrections, and other content not related to the research topic. The minimum number of occurrences of a term was selected as 10 and the most relevant term was chosen based on the relevance score. In this paper, 1600 terms were selected for inclusion in the glossary. In order to obtain a clearer visual description, less-used and irrelevant terms such as “age, year, etc.” were excluded, and the final number of relevant terms was determined to be 767.
(3) The title, authors, source, keywords, and abstract of the papers were screened. At this stage, papers that were not related to “community governance in the context of COVID-19” were excluded.
(4) In addition, articles were also excluded if they did not focus on social resilience in the COVID-19 context, or only used social resilience as a background for other activities; for example, just using the social vulnerability index (SVI) as a calculated metric to calculate metrics that evaluate other directions.
(5) The authors conducted content analysis on the selected papers. Statistical and quantitative analyses on the framework, research level, and evaluation indicators were undertaken. In addition, the policies, recommendations and implementations, and influencing factors of social resilience in the COVID-19 context were summarized. For policy, we divided its content into positive and negative aspects. Bruneau summarized resilience as having the characteristics of robustness, redundancy, rapidity, and resourcefulness (i.e., the 4R characteristics), which can be relied upon to reduce the impact of disasters and to recover more quickly to the original state, or an even better state, in the face of shocks and stresses [34]. This paper discerns how social resilience can help communities withstand COVID-19 by summarizing the four characteristics of social resilience based on the impact of recommendations and implementation.

## 3. Results

### 3.1. VOSviewer Visual Analysis of Social Resilience (2012–2022)

**Figure 1:**
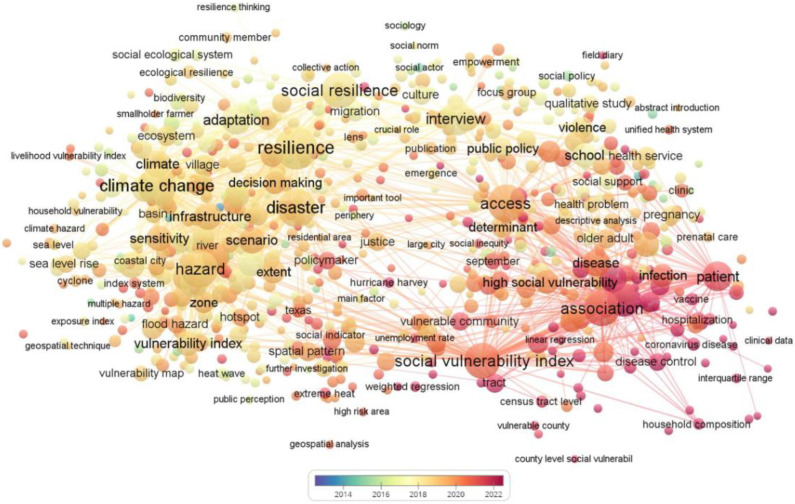
Visualization analysis of social resilience.

Figure 1 represents a visual analysis graph of the coverage of social resilience. From 2013 to 2015, research focused on exploring the concept of social resilience. For example, Keck proposed a definition of community resilience and summarized it in three dimensions by analyzing the existing literature [35]. From 2015 to 2017, scholars began to analyze social resilience impact factors and propose some assessment measures, starting from examples of damage caused by natural disasters to communities. Miao used the entropy method to analyze the recovery impacts of the Lushan earthquake on different affected communities and proposed feasible plans [36]. Between 2017 and 2018, research on social resilience transitioned from the unilateral impact of natural hazards to the impact of humans’ own participation. Aerts improved management policies by incorporating social and human behavior into flood risk assessment systems [37]. In 2018–2019, the direction of social resilience research began to shift towards analyzing the factors that influence social resilience and modeling the assessment framework. Rufat developed a social resilience vulnerability model and demonstrated its validity [38]. Since the outbreak of COVID-19 in 2019, research related to social resilience has increased each year as the community’s concern about the outbreak has grown, and resilience research has accounted for 20% of community resilience research from 2019 to July 2022.

### 3.2. Searching Process of Social Resilience (2019–2022)

**Figure 2:**
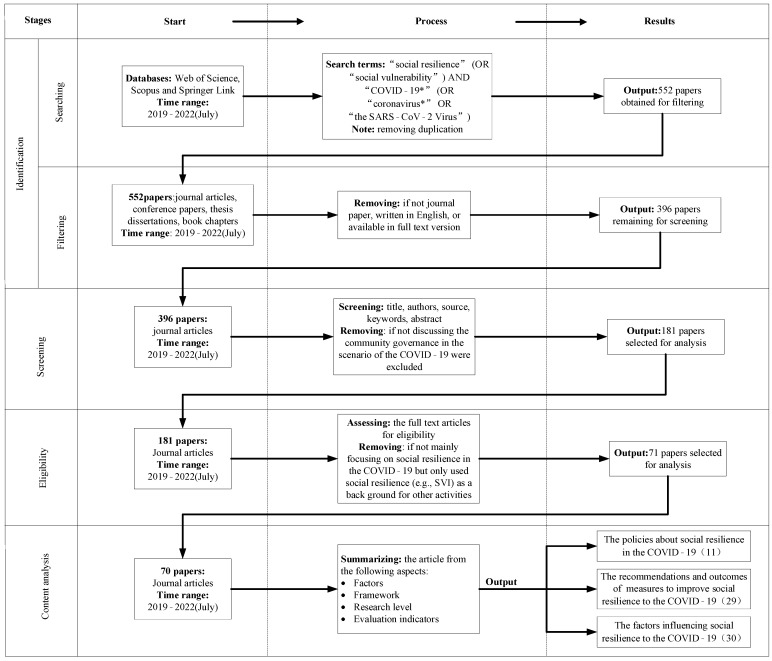
Flow diagram of the literature search and study selection.

The whole process includes four stages: Identification, Screening, Eligibility, and Content Analysis, as shown in Figure 2. Following the standards set above, 70 articles were finally chosen. Among them, 11 articles mentioned the policies relating to social resilience in COVID-19; 29 articles indicated the recommendations and measures to improve social resilience to COVID-19; and 30 articles emphasized the importance of the factors influencing social resilience to COVID-19.

### 3.3. The Policies about Social Resilience to the COVID-19

Around the world, governments have formulated different policies to deal with COVID-19, as shown in Table 1. Practice has proved that some policies are in line with national conditions and are very effective. For example, medical and non-medical measures are currently used by almost all governments. In contrast, some have had a negative impact on the country’s economic development and people’s well-being. For instance, the failure of some policies to take a long-term perspective has led to negative impacts on the lives and psyche of community residents, and a failure to consider whether policy implementation covers communities with less resilience.

### 3.4. The Recommendations and Measures of Social Resilience to the COVID-19

Robustness, redundancy, rapidity, and resourcefulness were introduced to classify scholars’ recommendations in terms of these four dimensions, as shown in Table 2.

Robustness as an outcome-oriented characteristic aims to enhance the strength of social resilience itself to withstand disaster impacts. For example, using culture and customs to enhance cohesion and organizational strength can improve the robustness of the community [50,51,52,53]; improving social health care coverage will improve social robustness [54,55]; and adaptive transformation of social capital helps to counteract the persistence of COVID-19 [56,57].

**Table 2:** The recommendations and measures of social resilience to COVID-19.

4R	Recommendations and Measures	Citation
Rapidity	Improve current “stay-at-home” policies that can have a psychiatric impact on community residents Focus on vulnerable groups and maintain appropriate neighborhood relations Establishing an URI (Urban Resilience Index)	[58,59,60,61]
Redundancy	Accessibility measures for COVID-19 patients need additional medical resources to improve Adopt comprehensive policies and measures to address substandard vaccine coverage for migrant workers and minorities Increasing vaccination rates among frontline workers	[62,63,64,65,66]
Resourcefulness	A strong system of local institutions working in concert with the state is needed to build a community-based, resilience-centered social resilience framework, a district-level CPVI, a conceptual model of CHASMS Government recommended to use digital telemedicine divide to address barriers to online treatment The importance of monitoring society through community questionnaires to prevent future COVID-19 transmission Government needs targeted vaccines for strategic vaccination to reduce inequities (focus on older populations), active advocacy and increased trust in vaccines among groups hesitant to vaccinate	[67,68,69,70,71,72,73,74,75]
Robustness	Improving coverage of emergency treatment response calls for low social resilience Governments need to balance policy and social side effects to strengthen the resilience of the system and facilitate the process of modeling the socio-spatial structure of urban space Prioritize public health and public support to control COVID-19 when chronic disease and COVID-19 coexist People’s daily lives have changed, and social sharing platforms should learn from each other about effective COVID-19 responses to provide more sustainable consumption and production patterns Social practice can strengthen community beliefs, improve collective effectiveness, and increase the strength of social resilience	[50,51,52,53,54,55,56,57,76,77,78]

Redundancy is the extent to which elements or other units of analysis exist that are substitutable and capable of satisfying functional requirements in the event of disruption, degradation, or loss of functionality [34]. It is the ability to replenish and maintain the necessary normal operations in the face of damage caused by a disaster. For example, it is important to distribute the vaccine, which is currently not abundant, to front-line and vulnerable people to ensure proper functioning and effective distribution of the vaccine in the face of the COVID-19 infection [62,63].

Resourcefulness meant to make society resilient and prepared to recover in the face of disasters. For instance, building an evaluation framework to assess social resilience in advance so that resources can be efficiently allocated to achieve resilience in the next COVID-19 outbreak [67,68,69,70,71], or pre-distributing the vaccines appropriately to enhance the resistance of vulnerable groups to COVID-19 [72,73,74].

Rapidity is often more responsive to the level of solidarity in a community. For example, the “stay-at-home” policy reflects how organized a community is and how quickly the policy is implemented, and that the community needs to be able to respond quickly to the policy [58,59].

From the perspective of the community, increasing the social resilience helps to control the spread of COVID-19.

(1) Robustness generally starts with community health services, organizations and community solidarity to improve their resistance to COVID-19;
(2) Redundancy is measured in terms of medical coverage, vaccination rates, and the distribution of vulnerable populations that are able to maintain good life-sustaining treatment and livelihoods despite exposure to COVID-19;
(3) Resourcefulness starts with the resilience framework and evaluation system, in addition to scientific and technological support, to grasp the overall situation of the whole community at a macro level and facilitate the development of policies;
(4) Rapidity is coupled with resourcefulness to enhance the community’s mastery of information, the rapid promulgation of policies, and the rational deployment of resources; these work together to achieve effective prevention and rapid control of COVID-19.

### 3.5. The Factors Influencing Social Resilience to the COVID-19

Thirty studies on the factors of social resilience to COVID-19 were identified, as shown in Table 3. The study of the influences of vulnerable groups accounted for 33% of these; the study of spatial variability, i.e., the study of the strength of resilience to epidemics in different communities, accounted for 27%; the analysis of psychosocial factors accounted for 13%; and the rest focused on neighborhoods, community services, and community capital.

Vulnerable populations are one of the most critical elements of social resilience to COVID-19. Vulnerable populations in the community are important contributors to increased COVID-19 infection rates, including the elderly, adolescents, disability groups, and refugees (or international migrants); improving social resilience to COVID-19 requires going back to the root causes to help vulnerable populations address their problems, including psychological and physical issues [79,80,81,82,83,84,85,86,88].

Spatial heterogeneity is the main factor that makes communities differ in their resilience. From a spatial and temporal perspective, analysis of areas with concentrated COVID-19 infection rates showed that areas with poor social resilience also had high infection rates, but areas with strong social resilience could also have high infection rates if they were not able to fulfill policies well or allocate resources appropriately [89,90,91,92,93,94,95,96].

The residents in the community themselves will in turn influence social resilience. Fitzpatrick et al. argued that psychological factors of community members have an impact on the maintenance of social resilience against COVID-19. Outbreaks that cause people to have life problems negatively affect community members; attention should be paid to the psychology of community members and appropriate adjustments should be made in time to ensure the robustness of resilience [97,98,99,100]. Some scholars focused on neighborhood relations between communities, and effective and harmonious neighborhood communication enhances the solidity of resilience against COVID-19 [105,106,108].

Uncovering the factors influencing social resilience to COVID-19 is actually a matter of finding the threats or causes of social resilience. From the above, it can be concluded that the individual behavior of residents in a community can have an impact on social resilience. The disproportionate impact of vulnerable groups on social resilience to COVID-19 contributes to the spatial heterogeneity of social resilience to epidemics. Secondly, the psychological behavior of community residents, economic sources, and mutual assistance among neighbors also influences social resilience to COVID-19.

## 4. Discussion

The previous research on COVID-19 social resilience generally considered two perspectives, the first through external interventions, and the second through internal regulation.

The regulation of social resilience requires external interventions, which can be divided into hard and soft power. The relationship between the two is complementary. From the studies reviewed, it appears that policy strength, infrastructure status, capital, and regulatory capability have been effective in controlling the spread of COVID-19 and reducing infection rates, thus ensuring that societies can maintain their resilience and learn from each other through tough policy. This allows the community to form a whole to better implement policies and respond in a timely and effective manner. However, these hardline interventions are difficult to maintain or enforce in less socially resilient areas and are poorly considered for vulnerable populations. For example, “stay-at-home” orders have little short-term impact on highly resilient societies with higher levels of capital; however, for less socially resilient areas, people have to go against the policy in order to make ends meet, leading to higher rates of infection.

In this case, soft power can be a good way to address the shortcomings. Using the cultural characteristics of the society, the level of public trust in each other and in the society can improve the overall solidarity and organizational capacity of the society, further ensuring that hard power, such as social policies, infrastructure, and medical services can sustain long-term operations during the COVID-19 pandemic. Interactions and permeability between societies can help increase the resilience of low societies.

Internal regulation ensures that social resilience is maintained. The natural conditions may result in spatial heterogeneity, making the distribution of social resilience inconsistent, while the human conditions are the basis for supporting social resilience. By analyzing the factors of population density, education level, language communication, and the proportion of vulnerable groups in a society, we can help the society itself to effectively assess, predict, and adjust its resilience and mitigate the impact of disasters. Discussing the state of survival and disease issues, and forming a complete database, ensures that society can remain adaptable and helps it allocate resources appropriately. This allows social resilience to be fundamentally enhanced and strengthens the ability of society as a whole to be able to withstand and rapidly recover from an epidemic or disaster. Within society, vulnerable groups have a greater impact on the resilience of the society. Vulnerable groups themselves are weaker in their perceptions and actions towards society. This is the target that must be focused on and on which protection is exerted. Vulnerable groups can be helped to provide better help and improve social resilience.

In this paper, the two research perspectives were divided into four dimensions and 20 indicators, as shown in Table 4.

## 5. Conclusions

This study reviewed and summarized articles on social resilience using a literature review. First, the current progress of research on social resilience was reviewed using VOSviewer visual analysis, and statistics showed that the number of publications on epidemic resilience increased in the past two years. Second, 70 papers on social resilience on COVID-19 were selected as the data, and the current fragmented reviews were categorized according to three aspects: analysis of existing policies, opinions and measures to enhance social resilience to the epidemic, and factors affecting social resilience to the epidemic. These papers were then grouped into three parts, and each part was classified and reviewed. Finally, a systematic framework was discussed to provide suggestions for integrating the literature on social resilience.

(1) After the COVID-19 pandemic, many communities incurred unrecoverable losses, and there was a purposeful effort to combine social resilience with epidemic preparedness, focusing on major public health events as a social resilience direction to promote the expansion of the social resilience field.
(2) Existing policies must be able to effectively control the spread of COVID-19, and the inadequacies of the policies should be investigated and evaluated, with additional recommendations and measures based on the resilience characteristics that need to be enhanced. Governments need to recognize that COVID-19 will be a near-term, ongoing epidemic, and need to take a long-term, dynamic approach to policy development. Furthermore, larger influences should be identified to fundamentally improve social resilience to COVID-19.
(3) Increasing the strength and regulatory precision of social resilience requires both external intervention and its own regulation. External hard interventions of policy, capital, and health care coverage are complemented by moderate healing, such as public trust and sociocultural characteristics. This can keep the level of social resilience within a manageable range and help society to control COVID-19 on a large scale. Thus, social resilience can be gradually increased at a reasonable rate. The self-regulation of natural and human conditions can fundamentally produce positive improvements in social resilience. In particular, the regulation of the human condition serves as the basis for the regulation of social resilience. It also focuses on larger influencing factors such as vulnerable groups and spatial heterogeneity to solidify the social resilience against COVID-19 from the “ground up”. People can learn from each other to help each other and gradually become connected to form urban resilience, and eventually achieve national consolidation and stability.

Despite the contribution to the research of social resilience on COVID-19, several limitations of this study need to be acknowledged. This research restricted the search of articles to English journal articles in Web of Science, Scopus, and Springer databases. There may be a number of other database or articles in other languages that can increase the availability of this research. Expanding the survey scope of the literature is suggested. More databases can be used to collect articles related to this topic, and non-English papers can be collected to make the study more robust. A more comprehensive survey can help future researchers explore more research possibilities.

Generally, the situation caused by the COVID-19 pandemic has created an opportunity to examine solutions and strategies to build and strengthen resilience-oriented planning in the direction of the community, starting with important factors, such as vulnerable groups, to address the impact of the pandemic and increase social resilience.

## Figures and Tables

**Table 1:** Policies related to social resilience to COVID-19.

Related Policies	Effects (Positive +/Negative −)	Citation
Social Distancing Policy	(+) Internal regulatory policy restrictions at the national level have inhibited the spread of COVID-19.	[39]
Mitigation Actions	(+) Mitigation policies can effectively control the spread of the epidemic	[40]
National Prevention Policies	(+) Policies (masks, home orders, congregation restrictions, social distance) had a significantly lower average mortality rate for low resilience communities.	[41]
	(+) Good political communication helps to improve political messages and thus social resilience.	[42]
COVID-19 policies applied to disabled people	(−) Policy responses should focus on social resilience and disabling barriers that force disabled people into states of vulnerability.	[43]
COVID-19 vaccination	(−) Vaccination resources are allocated with attention to community-level adaptation to ensure rationalization of vaccine supply.	[44]
Urban medical allocation policy	(−) Government action has resulted in an uneven distribution of the quality impact of access to the COVID-19 due to social inequalities at the individual and municipal levels.	[45]
Non-pharmacological intervention policies	(−) Many of these measures are not feasible for people living in socially maladjusted areas.	[46]
Stay-at-home	(−) Difficulties in complying with policies during “stay-at-home” periods are associated with less social resilience, maintaining it for a long time will have a negative impact on the psychology of the public.	[47,48]
The lockdowns	(−) COVID-19 exposed Australia’s systemic, demographic, and spatial vulnerabilities, while embargo policies led to reduced economic resilience.	[49]

**Table 3:** The factors influencing social resilience to COVID-19.

Influencing Factors and Percentage	Main Conclusions	Citation
Vulnerable Groups (33%)	Vulnerable groups as a key factor affecting social resilience during COVID-19 Patients living in resource-poor areas, especially among the elderly, children, minorities, homeless, and disadvantaged groups without transportation, expose public management practices and structures to higher rates of morbidity and mortality Middle-class and even affluent areas that lack the space, capital, and governance resilience can become “vulnerable” to COVID-19 Social resilience changes when refugees integrate into a new social system, and trauma-tized refugees are at increased risk of COVID-19 infection due to severe distrust and dis-respect for aid agencies, in addition to inadequate building infrastructure and unsanitary living conditions	[79,80,81,82,83,84,85,86,87,88]
Spatial Heterogeneity (27%)	Spatial heterogeneity of social resilience indicators (lower social resilience and lack of policies) was highly correlated with the spread of COVID-19 Socioeconomic status, racial/minority status, family composition, and environmental fac-tors are associated with COVID-19 morbidity and mortality Hot spots range from states with high percentages of densely populated and socially vul-nerable populations, to states with lax policy requirements and states with low vaccina-tion rates The impact of COVID-19 is dynamic, spreading first to large urban centers and more de-veloped cities, then to smaller and less developed cities, and then shifting back again	[89,90,91,92,93,94,95,96]
Social Psychological (13%)	The spread of COVID-19 has a negative psychological impact on people and seriously af-fects social resilience Female groups are more vulnerable to psychosocial and organizational pressures due to the economic and employment problems during the pandemic Social psychology is central to helping us understand the impact and resolution of COVID-19 on social attitudes and behaviors	[97,98,99,100]
Socioeconomic (13%)	Socioeconomic factors are strong predictors of COVID-19 outcomes, with housing density, the Municipal Human Development Index (MHDI) and SVI being the most influential fac-tors, and class segregation being a greater threat to the social and economic resilience Socioeconomic inequalities contribute to disparities in the COVID-19 population, espe-cially among children Cities in developing countries have weak social resilience due to lack of adequate facilities	[101,102,103,104]
Social Ties (7%)	Resilient communities have much lower case-fatality rates of COVID-19, and the most- and least-resilient groups in the community are prone to interact with communities similar to theirs, with increased mortality once the disease invades	[105,106]
Social Capital (3%)	Social capital helps residents adopt new behavioral norms	[107]
Social Assistance (4%)	Patients with poor social resilience were sicker, but with no difference in mortality or discharge disposition after hospital admission	[108]

**Table 4:** Division of research perspectives of social resilience to COVID-19.

Levels	Dimensions	Indicators	Explanations
Intervention Angle	Hard strength	Development Capacity	Social development adjustments affected by COVID-19
		Capital	The current economic state, ensuring redundancy in the economic aspects of resilience
		Policy Strength	Scope of policy enactment and effectiveness of governance
		Regulatory Capability	The extent to which policy regulates and can intervene
		Infrastructure Status	State and distribution of the social own infrastructure
		Medical Coverage	Social paramedical aid available
		Vaccinations	Vaccination status and distribution
	Soft Power	Public Assistance	Distribution of community benefit organizations and other service categories
		Neighborhood	The degree of harmony and organization among neighbors
		Trust Level	The degree of trust citizens has in a policy reflects the degree of willingness to implement it
		Cultural Resonance	Some positive customs that have a cohesive effect
		Security and Equity	Equitable and secure human survival
Internal Regulation	Natural Conditions	Natural Conditions	The physical geography in which the community itself is located
	Human conditions	Living Systems	The impact of the social labor production and living conditions
		Education level	Percentage of population with educational attainment
		Population Density	The average number of people on a certain unit of land at a certain time
		Percentage of vulnerable Groups	Percentage of vulnerable groups as a whole
		Diseases	Whether the individual has been ill during the epidemic and whether he or she has had other prior medical conditions such as chronic diseases
		Language Communication	A person’s language situation and ability to communicate properly with that community
		Mental state	Psychological situation of individuals during COVID-19

## Data Availability

The data presented in this study are available on request from the corresponding author. The data are not publicly available due to privacy.

## References

[1] Yang C., Sha D., Liu Q., Li Y., Lan H., Guan W.W., Hu T., Li Z., Zhang Z., Thompson J.H. (2020). Taking the pulse of COVID-19: A spatiotemporal perspective. Int. J. Digit. Earth.

[2] Orea L., Álvarez I.C. (2020). How effective has been the Spanish lockdown to battle COVID-19? A spatial analysis of the coronavirus propagation across provinces. Health Econ..

[3] Giuliani D., Dickson M.M., Espa G., Santi F. (2020). Modelling and Predicting the Spatio-Temporal Spread of Coronavirus Disease 2019 (COVID-19) in Italy. BMC Infect. Dis..

[4] Arab-Mazar Z., Sah R., Rabaan A.A., Dhama K., Rodriguez-Morales A.J. (2020). Mapping the incidence of the COVID-19 hotspot in Iran—Implications for Travellers. Travel Med. Infect. Dis..

[5] Franch-Pardo I., Napoletano B.M., Rosete-Verges F., Billa L. (2020). Spatial analysis and GIS in the study of COVID-19. A review. Sci. Total Environ..

[6] Elavarasan R.M., Pugazhendhi R., Shafiullah G.M., Irfan M., Anvari-Moghaddam A. (2021). A hover view over effectual approaches on pandemic management for sustainable cities-The endowment of prospective technologies with revitalization strategies. Sustain. Cities Soc..

[7] Mostafa M.K., Gamal G., Wafiq A. (2021). The impact of COVID 19 on air pollution levels and other environmental indicators—A case study of Egypt. J. Environ. Manag..

[8] Chirwa G.C., Dulani B., Sithole L., Chunga J.J., Alfonso W., Tengatenga J. (2022). Malawi at the Crossroads: Does the Fear of Contracting COVID-19 Affect the Propensity to Vote?. Eur. J. Dev. Res..

[9] Makhmudov N., Alisherovna A.G., Kazakov A. (2020). Analysis of the effect of coronavirus (COVID-19) on the development of the world economic system. Int. J. Integr. Educ..

[10] Coppola F., Faggiono L., Neri E., Grassi R., Miele V. (2021). Impact of the COVID-19 outbreak on the profession and psychological wellbeing of radiologists: A nationwide online survey. Insights into Imaging.

[11] Vollono C., Rollo E., Romozzi M., Frisullo G., Servidei S., Borghetti A., Calabresi P. (2020). Focal status epilepticus as unique clinical feature of COVID-19: A case report. Seizure Eur. J. Epilepsy.

[12] Kresch E., Achua J., Saltzman R., Khodamoradi K., Arora H., Ibrahim E., Kryvenko O.N., Almeida V.W., Firdaus F., Hare J.M. (2021). COVID-19 Endothelial Dysfunction Can Cause Erectile Dysfunction: Histopathological, Immunohistochemical, and Ultrastructural Study of the Human Penis. World J. Mens Health.

[13] Werthman-Ehrenreich A. (2021). Mucormycosis with orbital compartment syndrome in a patient with COVID-19. Am. J. Emerg. Med..

[14] Goldstein J.R., Lee R.D. (2020). Demographic perspectives on the mortality of COVID-19 and other epidemics. Proc. Natl. Acad. Sci. USA.

[15] Tönnies F., Loomis C.P. (1963). Community and Society.

[16] Deng P., Ouyang Y. Research on the Method of Improving Community Governance Capability Based on Block-Chain Technology. Proceedings of the 2021 IEEE International Conference on Consumer Electronics and Computer Engineering (ICCECE).

[17] Van Belle S., Affun-Adegbule C., Soors W., Srinivas P.N., Hegel G., Van Damme W., Saluja D., Abejirinde I., Wouters E., Masquillier C. (2020). COVID-19 and informal settlements: An urgent call to rethink urban governance. Int. J. Equity Health.

[18] Gu E., Li L. (2020). Crippled community governance and suppressed scientific/professional communities: A critical assessment of failed early warning for the COVID-19 outbreak in China. J. Chin. Gov..

[19] Zaman S., Hossain F., Matin I. (2022). Ethnography of community governance: A case of COVID-19 response of an urban slum in Bangladesh. Community Dev. J..

[20] Wang F., Fang Y.Y., Deng H.D., Wei F.Z. (2022). How community medical facilities can promote resilient community constructions under the background of pandemics. Indoor Built Environ..

[21] Cui J., Liu X. (2021). Study on Community Integration and Community Governance of Korean Residents in Tianjin International Community Amid the COVID-19 Pandemic. Stud. Koreans Abroad.

[22] Turner B.L. (2010). Vulnerability and resilience: Coalescing or paralleling approaches for sustainability science?. Glob. Environ. Chang. Hum. Policy Dimens..

[23] Klein R.J.T., Nicholls R., Thomalla F.J. (2003). Hazards, Resilience to natural hazards: How useful is this concept?. Glob. Environ. Chang. Part B Environ. Hazards.

[24] Béné C., Evans L., Mills D., Ovie S., Raji A., Tafida A., Kodio A., Sinaba F., Morand P., Lemoalle J. (2011). Testing resilience thinking in a poverty context: Experience from the Niger River basin. Glob. Environ. Chang. Hum. Policy Dimens..

[25] Davis R., Cook D., Cohen L. (2005). A community resilience approach to reducing ethnic and racial disparities in health. Am. J. Public Health.

[26] Kong L.M., Zhang L., Ye L.J., Yang J.W., Liu Y.L., Song S.L. (2022). To take the initiative in the prevention and control of the coronavirus disease 2019 epidemic in the changing and unchanged strategies. Zhonghua Yi Xue Za Zhi.

[27] Moya J., Goenechea M. (2022). An Approach to the Unified Conceptualization, Definition, and Characterization of Social Resilience. Int. J. Environ. Res. Public Health.

[28] Liu Z.L., Lin S.N., Lu T.T., Shen Y., Liang S.S. (2022). Towards a constructed order of co-governance: Understanding the state-society dynamics of neighbourhood collaborative responses to COVID-19 in urban China. Urban Stud..

[29] Remuzzi A., Remuzzi G. (2020). COVID-19 and Italy: What next?. Lancet.

[30] Aldhahi M.I., Akil S., Zaidi U., Mortada E., Award S., Al Awaji N. (2021). Effect of Resilience on Health-Related Quality of Life during the COVID-19 Pandemic: A Cross-Sectional Study. Int. J. Environ. Res. Public Health.

[31] Myers N. (2021). Information Sharing and Community Resilience: Toward a Whole Community Approach to Surveillance and Combatting the “Infodemic”. World Med. Health Policy.

[32] Ranjbari M., Esfandabadi Z.S., Zanetti M.C., Scagnelli S.D., Siebers P.O., Aghbashlo M., Peng W.X., Quatraro F., Tabatabaei M. (2021). Three pillars of sustainability in the wake of COVID-19: A systematic review and future research agenda for sustainable development. J. Clean. Prod..

[33] Moher D., Liberati A., Tetzlaff J., Altman D.G. (2010). Preferred Reporting Items for Systematic Reviews and Meta-Analyses: The PRISMA Statement. Int. J. Surg..

[34] Bruneau M., Chang S.E., Eguchi R.T., Lee G.C., O’Rourke T.D., Reinhorn A.M., Shinozuka M., Tierney K., Wallace W.A., Von Winterfeldt D. (2019). A Framework to Quantitatively Assess and Enhance the Seismic Resilience of Communities. Earthq. Spectra.

[35] Keck M., Sakdapolrak P. (2013). What is social resilience? Lessons learned and ways forward. Erdkunde.

[36] Miao C., Ding M.T. (2015). Social vulnerability assessment of geological hazards based on entropy method in Lushan earthquake-stricken area. Arab. J. Geosci..

[37] Aerts J., Botzen W.J., Clarke K.C., Cutter S.L., Hall J.W., Merz B., Michel-Kerjan E., Mysiak J., Surminski S., Kunreuther H. (2018). Integrating human behaviour dynamics into flood disaster risk assessment. Nat. Clim. Chang..

[38] Rufat S., Tate E., Emrich C.T., Antolini F. (2019). How Valid Are Social Vulnerability Models?. Ann. Am. Assoc. Geogr..

[39] Luo Z.W., Li L., Ma J.F., Tang Z., Shen H., Zhu H.H., Wu B. (2022). Moderating Effect of a Cross-Level Social Distancing Policy on the Disparity of COVID-19 Transmission in the United States. ISPRS Int. J. Geo Inf..

[40] Huang Q., Jackson S., Derakhshan S., Lee L., Pham E., Jackson A., Cutter S.L. (2021). Urban-rural differences in COVID-19 exposures and outcomes in the South: A preliminary analysis of South Carolina. PLoS ONE.

[41] Page-Tan C., Corbin T.B. (2021). Protective policies for all? An analysis of COVID-19 deaths and protective policies among low-, medium-, and high-vulnerability groups. Disasters.

[42] Fernandez-Prados J.S., Lozano-Diaz A., Muyor-Rodriguez J. (2021). Factors explaining social resilience against COVID-19: The case of Spain. Eur. Soc..

[43] Mladenov T., Brennan C.S. (2021). Social vulnerability and the impact of policy responses to COVID-19 on disabled people. Sociol. Health Illn..

[44] Hughes M.M., Wang A.L.C., Grossman M.K., Pun E., Whiteman A., Deng L., Hallisey E., Shape J.D., Ussery E.N., Stokley S. (2021). County-Level COVID-19 Vaccination Coverage and Social Vulnerability—United States, December 14, 2020–March 1, 2021. MMWR Morb. Mortal. Wkly. Rep..

[45] Antonio-Villa N.E., Fernandez-Chirino L., Pisanty-Alatorre J., Mancilla-Galindo J., Kammar-Garcia A., Vargas-Vazquez A., Gonzalez-Diaz A., Fermin-Martinez C.A., Marquez-Salinas A., Guerra E.C. (2022). Comprehensive Evaluation of the Impact of Sociodemographic Inequalities on Adverse Outcomes and Excess Mortality During the Coronavirus Disease 2019 (COVID-19) Pandemic in Mexico City. Clin. Infect. Dis..

[46] Raymundo C.E., Oliveira M.C., Eleuterio T.D., Andre S.R., da Silva M.G., Queiroz E.R.D., Medronho R.D. (2021). Spatial analysis of COVID-19 incidence and the sociodemographic context in Brazil. PLoS ONE.

[47] Ferreira R.J., Buttell F., Cannon C. (2020). COVID-19: Immediate Predictors of Individual Resilience. Sustainability.

[48] Fletcher K.M., Espey J., Grossman M.K., Sharpe J.D., Curriero F.C., Wilt D.E., Sunshine G., Moreland A., Howard-Williams M., Ramos J.G. (2021). Social vulnerability and county stay-at-home behavior during COVID-19 stay-at-home orders, United States, April 7-April 20, 2020. Ann. Epidemiol..

[49] Karacsonyi D., Dyrting S., Taylor A. (2021). A spatial interpretation of Australia’s COVID-vulnerability. Int. J. Disaster Risk Reduct..

[50] Fatmawati, Dewantara J.A. (2022). Social resilience of indigenous community on the border: Belief and confidence in anticipating the spread of COVID-19 through the Besamsam custom in the Dayak community. J. Community Appl. Soc. Psychol..

[51] Popa N., Pop A.M., Marian-Potra A.C., Cocean P., Hognogi G.G., David N.A. (2021). The Impact of the COVID-19 Pandemic on Independent Creative Activities in Two Large Cities in Romania. Int. J. Environ. Res. Public Health.

[52] Minguez Garcia B. (2021). Integrating culture in post-crisis urban recovery: Reflections on the power of cultural heritage to deal with crisis. Int. J. Disaster Risk Reduct..

[53] Valizadeh N., Ghazani E., Akbari M., Shekarkhah J. (2022). How Do Collective Efficiency and Norms Influence the Social Resilience of Iranian Villagers Against the COVID-19? The Mediating Role of Social Leadership. Front. Public Health.

[54] Duarte R., Aguiar A., Pinto M., Furtado I., Tiberi S., Lonnroth K., Migliori G.B. (2021). Different disease, same challenges: Social determinants of tuberculosis and COVID-19. Pulmonology.

[55] Haas E.J., Furek A., Casey M., Yoon K.N., Moore S.M. (2021). Applying the Social Vulnerability Index as a Leading Indicator to Protect Fire-Based Emergency Medical Service Responders’ Health. Int. J. Environ. Res. Public Health.

[56] Mont O., Curtis S.K., Voytenko Palgan Y. (2021). Organisational Response Strategies to COVID-19 in the Sharing Economy. Sustain. Prod. Consum..

[57] Douglas M., Katikireddi S.V., Taulbut M., McKee M., McCartney G. (2020). Mitigating the wider health effects of COVID-19 pandemic response. BMJ Br. Med. J..

[58] Fu X.Y., Zhai W. (2021). Examining the spatial and temporal relationship between social vulnerability and stay-at-home behaviors in New York City during the COVID-19 pandemic. Sustain. Cities Soc..

[59] Ulimwengu J., Kibonge A. (2021). Spatial spillover and COVID-19 spread in the US. BMC Public Health.

[60] Xiong J.Q., Lipsitz O., Nasri F., Lui L.M.W., Gill H., Phan L., Chen-Li D., Iacobucci M., Ho R., Majeed A. (2020). Impact of COVID-19 pandemic on mental health in the general population: A systematic review. J. Affect. Disord..

[61] Chen Y., Su X.Y., Zhou Q. (2021). Study on the Spatiotemporal Evolution and Influencing Factors of Urban Resilience in the Yellow River Basin. Int. J. Environ. Res. Public Health.

[62] Kang J.Y., Michels A., Lyu F.Z., Wang S.H., Agbodo N., Freeman V.L., Wang S.W. (2020). Rapidly measuring spatial accessibility of COVID-19 healthcare resources: A case study of Illinois, USA. Int. J. Health Geogr..

[63] Mofleh D., Almohamad M., Osaghae I., Bempah S., Zhang Q.Z., Tortolero G., Ebeidat A., Ramphul R., Sharma S.V. (2022). Spatial Patterns of COVID-19 Vaccination Coverage by Social Vulnerability Index and Designated COVID-19 Vaccine Sites in Texas. Vaccines.

[64] Gharpure R., Yi S.H., Li R.R., Slifka K.M.J., Tippins A., Jaffe A., Guo A., Kent A.G., Gouin K.A., Whitworth J.C. (2021). COVID-19 Vaccine Uptake Among Residents and Staff Members of Assisted Living and Residential Care Communities-Pharmacy Partnership for Long-Term Care Program, December 2020–April 2021. J. Am. Med. Dir. Assoc..

[65] Sohn H., Aqua J.K. (2022). Geographic variation in COVID-19 vulnerability by legal immigration status in California: A prepandemic cross-sectional study. BMJ Open.

[66] Blukacz A., Cabieses B., Mezones-Holguin E., Arias J.M.C. (2022). Healthcare and social needs of international migrants during the COVID-19 pandemic in Latin America: Analysis of the Chilean case. Glob. Health Promot..

[67] Mouliou D.S., Kotsiou O.S., Gourgoulianis K.I. (2021). Estimates of COVID-19 Risk Factors among Social Strata and Predictors for a Vulnerability to the Infection. Int. J. Environ. Res. Public Health.

[68] Gupta D., Fischer H., Shrestha S., Ali S.S., Chhatre A., Devkota K., Fleischman F., Khatri D.B., Rana P. (2021). Dark and bright spots in the shadow of the pandemic: Rural livelihoods, social vulnerability, and local governance in India and Nepal. World Dev..

[69] Zhang F.X. (2022). The community resilience measurement throughout the COVID-19 pandemic and beyond -an empirical study based on data from Shanghai, Wuhan and Chengdu. Int. J. Disaster Risk Reduct..

[70] Thomas D.S.K., Jang S., Scandlyn J. (2020). The CHASMS conceptual model of cascading disasters and social vulnerability: The COVID-19 case example. Int. J. Disaster Risk Reduct..

[71] Saghapour T., Giles-Corti B., Jafari A., Qaisrani M.A., Turrell G. (2021). Supporting pandemic disease preparedness: Development of a composite index of area vulnerability. Health Place.

[72] Wang H., Xu R., Qu S.J., Schwatz M., Adams A., Chen X. (2021). Health inequities in COVID-19 vaccination among the elderly: Case of Connecticut. J. Infect. Public Health.

[73] Khairat S., Zou B.M., Adler-Milstein J. (2022). Factors and reasons associated with low COVID-19 vaccine uptake among highly hesitant communities in the US. Am. J. Infect. Control.

[74] Li Z., Lewis B., Berney K., Hallisey E., Williams A.M., Whiteman A., Rivera-Gonzalez L.O., Clarke K.E.N., Clayton H.B., Tincher T. (2022). Social Vulnerability and Rurality Associated With Higher Severe Acute Respiratory Syndrome Coronavirus 2 (SARS-CoV-2) Infection-Induced Seroprevalence: A Nationwide Blood Donor Study-United States, July 2020–June 2021. Clin. Infect. Dis..

[75] Fu H.L., Zhu H., Xue P.D., Hu X., Guo X.T., Liu B.S. (2022). Eye-tracking study of public acceptance of 5G base stations in the context of the COVID-19 pandemic. Eng. Constr. Archit. Manag..

[76] Wieringa S., Neves A.L., Rushforth A., Ladds E., Husain L., Finlay T., Pope C., Greenhalgh T. (2022). Safety implications of remote assessments for suspected COVID-19: Qualitative study in UK primary care. BMJ Qual. Saf..

[77] Islam N., Lacey B., Shabnam S., Erzurumluoglu A.M., Dambha-Miller H., Chowell G., Kawachi I., Marmot M. (2021). Social inequality and the syndemic of chronic disease and COVID-19: County-level analysis in the USA. J. Epidemiol. Community Health.

[78] Galacho-Jimenez F.B., Carrunan-Herrera D., Molina J., Ruiz-Sinoga J.D. (2022). Evidence of the Relationship between Social Vulnerability and the Spread of COVID-19 in Urban Spaces. Int. J. Environ. Res. Public Health.

[79] Gaynor T.S., Wilson M.E. (2020). Social Vulnerability and Equity: The Disproportionate Impact of COVID-19. Public Adm. Rev..

[80] Prodanuk M., Wagner S., Orkin J., Noone D. (2021). Social vulnerability and COVID-19: A call to action for paediatric clinicians Comment. Paediatr. Child Health.

[81] Tummalapalli S.L., Silberzweig J., Cukor D., Lin J.T., Barbar T., Liu Y., Kim K., Parker T.S., Levine D.M., Ibrahim S.A. (2021). Racial and Neighborhood-Level Disparities in COVID-19 Incidence among Patients on Hemodialysis in New York City. J. Am. Soc. Nephrol..

[82] Akter S., Dhar T.K., Rahman A.I.A., Uddin M.K. (2021). Investigating the resilience of refugee camps to COVID-19: A case of Rohingya settlements in Bangladesh. J. Migr. Health.

[83] Martin-Sanchez F.J., Carbo A.V., Miro O., Llorens P., Jimenez S., Pinera P., Burillo-Putze G., Martin A., Garcia-Lamberechts J.E., Jacob J. (2021). Socio-Demographic Health Determinants Are Associated with Poor Prognosis in Spanish Patients Hospitalized with COVID-19. J. Gen. Intern. Med..

[84] Oates G.R., Juarez L.D., Horswell R., Chu S., Miele L., Fouad M.N., Curry W.A., Fort D., Hillegass W.B., Danos D.M. (2021). The Association Between Neighborhood Social Vulnerability and COVID-19 Testing, Positivity, and Incidence in Alabama and Louisiana. J. Community Health.

[85] De Souza C.D.F., Machado M.F., do Carmo R.F. (2020). Human development, social vulnerability and COVID-19 in Brazil: A study of the social determinants of health. Infect. Dis. Poverty.

[86] Karaye I.M., Horney J.A. (2020). The Impact of Social Vulnerability on COVID-19 in the US: An Analysis of Spatially Varying Relationships. Am. J. Prev. Med..

[87] Shi C.Y., Liao L., Li H., Su Z.H. (2022). Which urban communities are susceptible to COVID-19? An empirical study through the lens of community resilience. BMC Public Health.

[88] Falkenhain M., Flick U., Hirseland A., Naji S., Seidelsohn K., Verlage T. (2021). Setback in labour market integration due to the Covid-19 crisis? An explorative insight on forced migrants’ vulnerability in Germany. Eur. Soc..

[89] Troppy S., Wilt G.E., Whiteman A., Hallisey E., Crockett M., Sharpe J.D., Haney G., Cranston K., Klevens R.M. (2021). Geographic Associations Between Social Factors and SARS-CoV-2 Testing Early in the COVID-19 Pandemic, February–June 2020, Massachusetts. Public Health Rep..

[90] Neelon B., Mutiso F., Mueller N.T., Pearce J.L., Benjamin-Neelon S.E. (2021). Spatial and temporal trends in social vulnerability and COVID-19 incidence and death rates in the United States. PLoS ONE.

[91] Coelho F.C., Lana R.M., Cruz O.G., Villela D.A.M., Bastos L.S., Piontti A.P.Y., Davis J.T., Vespignani A., Codeco C.T., Gomes M.F.C. (2020). Assessing the spread of COVID-19 in Brazil: Mobility, morbidity and social vulnerability. PLoS ONE.

[92] Karmakar M., Lantz P.M., Tipirneni R. (2021). Association of Social and Demographic Factors With COVID-19 Incidence and Death Rates in the US. JAMA Netw. Open.

[93] Park Y.M., Kearney G.D., Wall B., Jones K., Howard R.J., Hylock R.H. (2021). COVID-19 Deaths in the United States: Shifts in Hot Spots over the Three Phases of the Pandemic and the Spatiotemporally Varying Impact of Pandemic Vulnerability. Int. J. Environ. Res. Public Health.

[94] Jackson S.L., Derakhshan S., Blackwood L., Lee L., Huang Q., Habets M., Cutter S.L. (2021). Spatial Disparities of COVID-19 Cases and Fatalities in United States Counties. Int. J. Environ. Res. Public Health.

[95] Baggio J.A.O., Machado M.F., do Carmo R.F., Armstrong A.D., dos Santos A.D., de Souza C.D.F. (2021). COVID-19 in Brazil: Spatial risk, social vulnerability, human development, clinical manifestations and predictors of mortality—A retrospective study with data from 59 695 individuals. Epidemiol. Infect..

[96] Johnson D.P., Ravi N., Braneon C.V. (2021). Spatiotemporal Associations Between Social Vulnerability, Environmental Measurements, and COVID-19 in the Conterminous United States. Geohealth.

[97] Fitzpatrick K.M., Harris C., Drawve G. (2020). How bad is it? Suicidality in the middle of the COVID-19 pandemic. Suicide Life Threat. Behav..

[98] Syed A.A., Gupta S., Rai D. (2021). Psychological, social and economic impact of COVID 19 on the working population of India: Exploratory factor analysis approach. Int. J. Disaster Risk Reduct..

[99] Krings V.C., Steeden B., Abrams D., Hogg M.A. (2021). Social attitudes and behavior in the COVID-19 pandemic: Evidence and prospects from research on group processes and intergroup relations. Group Process. Intergroup Relat..

[100] Salari N., Hosseinian-Far A., Jalali R., Vaisi-Raygani A., Rasoulpoor S., Mohammadi M., Rasoulpoor S., Kjaledi-Paveh B. (2020). Prevalence of stress, anxiety, depression among the general population during the COVID-19 pandemic: A systematic review and meta-analysis. Glob. Health.

[101] Bin Kashem S., Baker D.M., Gonzalez S.R., Lee C.A. (2021). Exploring the nexus between social vulnerability, built environment, and the prevalence of COVID-19: A case study of Chicago. Sustain. Cities Soc..

[102] Martins P.R., Quintans-Junior L.J., Araujo A.A.D., Sposato K.B., Tavares C.S.S., Gurgel R.Q., Leite D.C.F., de Paiva S.M., Santos H.P., Santos V.S. (2021). Socio-economic inequalities and COVID-19 incidence and mortality in Brazilian children: A nationwide register-based study. Public Health.

[103] Alizadeh H., Sharifi A. (2021). Analysis of the state of social resilience among different socio-demographic groups during the COVID- 19 pandemic. Int. J. Disaster Risk Reduct..

[104] Castro R.R., Santos R.S.C., Sousa G.J.B., Pinheiro Y.T., Martins R.R.I.M., Pereira M.L.D., Silva R.A.R. (2021). Spatial dynamics of the COVID-19 pandemic in Brazil. Epidemiol. Infect..

[105] Li X., Huang X., Li D.Y., Xu Y. (2022). Aggravated social segregation during the COVID-19 pandemic: Evidence from crowdsourced mobility data in twelve most populated US metropolitan areas. Sustain. Cities Soc..

[106] Fraser T., Aldrich D.P. (2021). The dual effect of social ties on COVID-19 spread in Japan. Sci. Rep..

[107] Fraser T., Page-Tan C., Aldrich D.P. (2022). Social capital’s impact on COVID-19 outcomes at local levels. Sci. Rep..

[108] Tipirneni R., Karmakar M., O’Malley M., Prescott H.C., Chopra V. (2022). Contribution of Individual- and Neighborhood-Level Social, Demographic, and Health Factors to COVID-19 Hospitalization Outcomes. Ann. Intern. Med..

